# A quantitative comparison of antibodies against epitope tags for immunofluorescence detection

**DOI:** 10.1002/2211-5463.13705

**Published:** 2023-11-03

**Authors:** Anna Marchetti, Wanessa C. Lima, Philippe Hammel, Pierre Cosson

**Affiliations:** ^1^ Department of Cell Physiology and Metabolism, Faculty of Medicine University of Geneva Switzerland

**Keywords:** 6xHis, ABCD database, Epitope tags, FLAG, HA, Myc

## Abstract

Epitope tags recognized by specific antibodies have been widely used over the last few decades, notably to localize tagged proteins within cells by immunofluorescence. The diversity of tags and antibodies usually prevents a side‐by‐side comparison of the efficiency with which each antibody recognizes its cognate tag. We expressed chimeric proteins, each composed of an invariant domain (IL2Ra) associated with a specific epitope tag. Double immunofluorescence allowed us to quantify in parallel the reference signal generated by the anti‐IL2Ra antibody and the signal generated by the anti‐epitope tag antibody. Since all antibodies used in this study were recombinant antibodies fused to the same mouse Fc domain, the generated signals were directly comparable. Three groups of tags/antibodies were revealed: ‘good’ antibodies generated high signals even when used at a low concentration (50 ng·mL^−1^), ‘fair’ antibodies generated a high signal only at high concentrations (5000 ng·mL^−1^), and ‘mediocre’ antibodies generated positive but weak signals. Except for an anti‐myc antibody, similar results were obtained when cells were fixed in paraformaldehyde or methanol. These results provide a side‐by‐side quantitative evaluation of different tag/antibody pairs. This information will be useful to optimize the choice of epitope tags and to choose optimal antibodies.

## Graphical abstract

In this study, we compared side‐by‐side and quantitatively a collection of epitope tags. We also determined what the best recombinant antibodies are to detect each epitope tag, and in which condition they can be used. This study will be useful for researchers using epitope tags and specific anti‐tag antibodies.

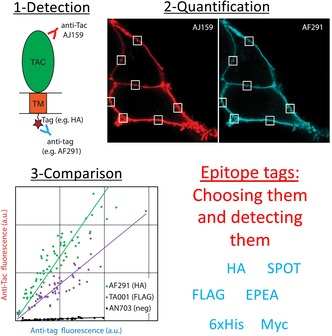

**Abbreviations**
HEK: human embryonic kidney
IgG: Immunoglobulin G
scFv: single‐chain variable fragment

The intracellular localization of a protein of interest can be determined by immunofluorescence in fixed and permeabilized cells using specific antibodies. However, for many proteins, no specific antibodies are available. An alternative strategy is to express a modified version of the protein of interest, where a short peptide sequence (usually referred to as an epitope tag) has been introduced, which is then recognized by a specific anti‐tag antibody. Epitope tagging was pioneered in 1984, using a short peptide from neuropeptide substance P and a monoclonal antibody specifically binding this sequence [1]. Since then, a wide collection of epitope/antibody pairs has been developed [2], including epitope tags derived from natural proteins (Myc, HA) [3, 4] or designed epitope tags (6xHis, DYKDDDDK) [5, 6]. DYKDDDDK is also known as the FLAG tag: FLAG^®^ is a registered trademark of Sigma‐Aldrich Biotechnology (Burlington, MA, USA). The monoclonal antibodies that were initially used to recognize epitope tags were later sequenced and can be produced today as recombinant antibodies. More recently, single‐domain camelid antibodies recognizing epitope tags (EPEA, SPOT) have been described [7]. Each epitope tag exhibits specific properties: size, hydrophilicity, presence of lysine residues (potentially sensitive to formaldehyde fixation), etc. In addition, different antibodies may recognize the same tag with different efficiencies. Finally, the recognition may be affected by the accessibility of the tag, the folding of the protein (e.g., denatured vs native), or the technique used (e.g., western blot vs immunofluorescence). It has long been recognized that some antibodies may not have an optimal affinity for the corresponding peptide tag. To circumvent this problem, some researchers have tagged their proteins with multiple copies of the same epitope tag [8]. However, the diversity of the situations did not allow a meaningful comparison of the different epitope/antibody pairs. In addition, several of these epitope/antibody pairs are patented and sold by private companies, a situation not ideal for unbiased comparisons. The aim of this study was to compare side‐by‐side the efficiency with which different tags are recognized by their cognate antibody during an immunofluorescence staining of fixed cells.

## Results

### A comparative assessment of different tags

**Fig. 1:**
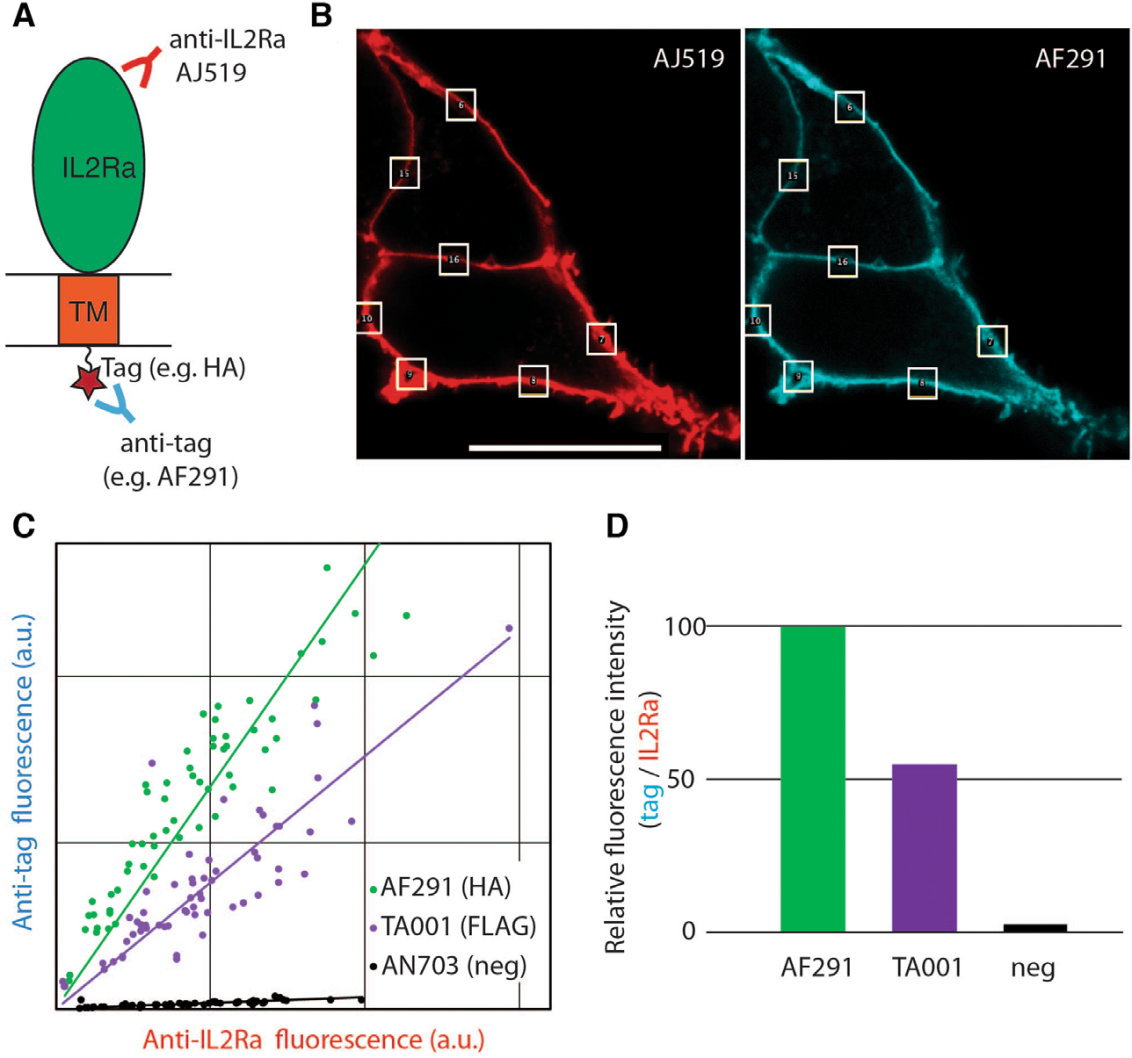
Strategy to determine the relative efficiency with which antibodies recognize their cognate tag. (A) Plasmids encoding a fusion protein composed of the IL2Ra extracellular and transmembrane domains fused to a cytosolic tag (e.g., HA) were transfected in HEK cells. (B) The IL2Ra‐tag fusion protein was detected with an anti‐IL2Ra antibody (AJ519, red) and an anti‐tag antibody (e.g., AF291, blue). The fluorescence levels were determined over a number of small areas (white boxes). Scale bar: 20 μm. (C) The fluorescence levels were plotted for each antibody, and linear regression was used to calculate the relative efficiency with which the anti‐tag recognized its epitope. (D) For each antibody, the relative binding efficiency was compared with the value obtained for the AF291 antibody (set to 100), included in all experiments.

In order to compare the efficiency with which different antibodies recognize their cognate tag, we expressed in human embryonic kidney (HEK) cells fusion proteins composed of the transmembrane α chain of the interleukin 2 receptor (IL2Ra, also known as the Tac antigen) [9] with a linear epitope tag at the C‐terminal end of its cytosolic domain (Fig. 1A). Six different tags were used: EPEA, DYKDDDDK (FLAG^®^), HA, 6xHis, Myc, and SPOT (Table 1). The cells were fixed with paraformaldehyde and permeabilized with saponin. The IL2Ra fusion protein was detected simultaneously with an anti‐IL2Ra antibody (AJ519, coupled to a rabbit Fc domain) [10] and a recombinant antibody recognizing the fused epitope tag (e.g., anti‐HA fused to a mouse Fc domain; Fig. 1B). All fusion proteins (e.g., IL2Ra‐HA) were detected almost exclusively at the cell surface, indicating that they were efficiently transported to the plasma membrane (data not shown). For each tag, we tested from one to three different recombinant antibodies (Table 1). The signals generated by each anti‐tag antibody and by the anti‐IL2Ra antibody were quantified over multiple small regions in each picture. The aim of this sampling method was to reduce the background by focusing the analysis on regions where the specific signal was high. For each region analyzed, the fluorescence in the two fluorescent channels was measured and plotted (Fig. 1C). A linear regression was then used to determine the ratio of the two signals, from which one can deduce the relative efficiency with which the anti‐IL2Ra and the anti‐tag antibodies recognized the same target protein (Fig. 1C). To obtain comparable results in independent experiments, one positive control (anti‐HA AF291) was included in every experiment and was attributed the arbitrary value of 100 (Fig. 1D).

**Table 1:** Epitope tags and recombinant antibodies used in this study; for each epitope, the best antibody identified in this study is underlined and in bold.

Epitope	Peptide sequence	Antibody	Fc domain	Reference
EPEA	GGEPEA	**AI215**	Mouse IgG2A	[12]
SPOT	PDRVRAVSHWSS	**AI196**	Mouse IgG2A	[13]
Myc	EQKLISEEDLL	AI179	Mouse IgG2A	[14]
		**TA002**		This study
HA	YPYDVPDYASLRS	**AF291**	Mouse IgG2A	[15]
6xHis	HHHHHH	AF371	Mouse IgG2A	[21]
		AD946		[6]
		**AV248**		[16]
Flag	DYKDDDDK	AI177	Mouse IgG2A	[17]
		AX047		[22]
		**TA001**		This study
Hs IL2Ra	Uniprot P01589	AJ519	Rabbit IgG	[10]
Dd PDI	Uniprot Q86IA3	AN703	Mouse IgG2A	[18]

### Antibodies recognize their cognate tag with different efficiencies

**Fig. 2:**
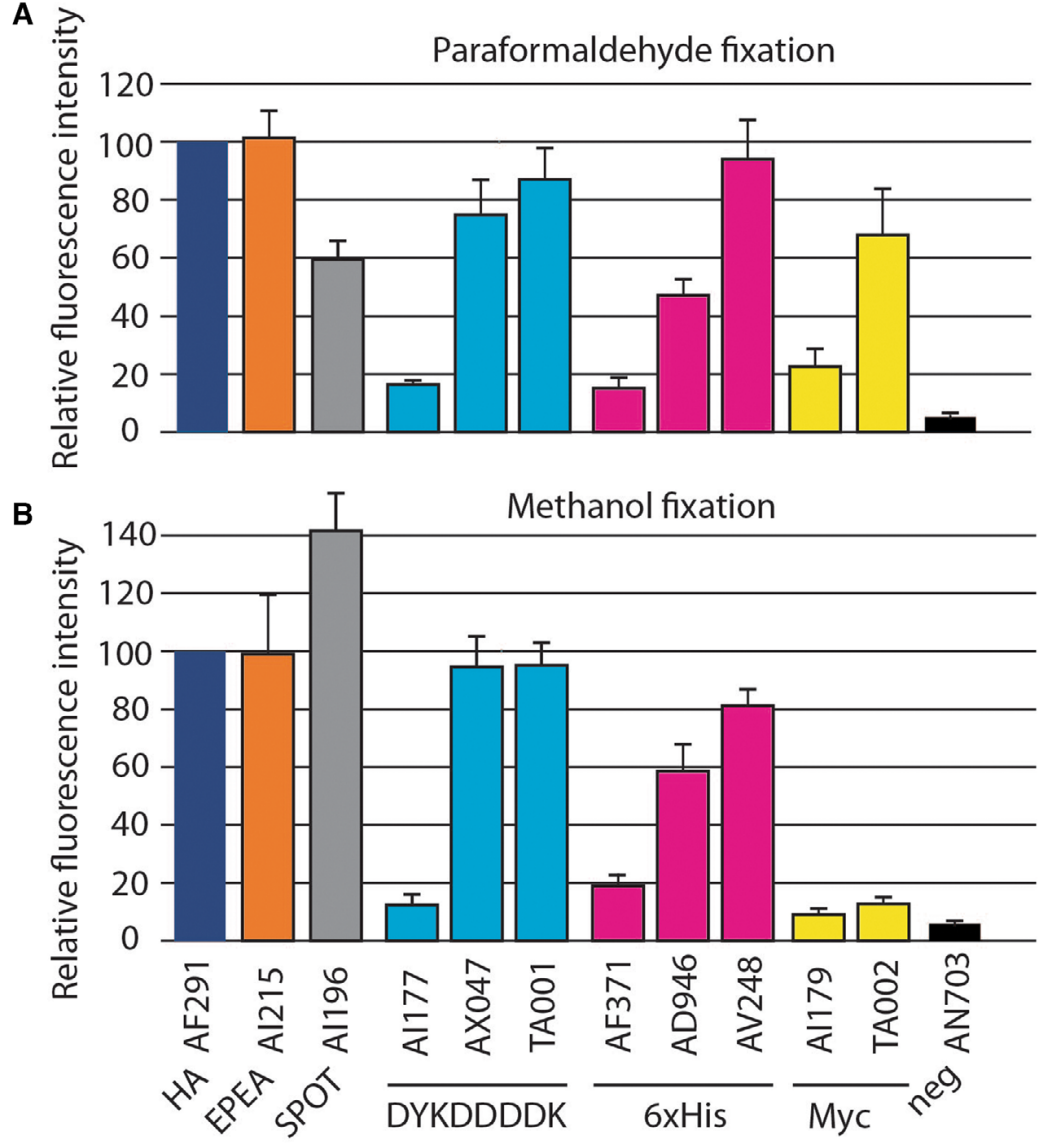
Quantification of the signals generated by different antibodies. The signal generated by each antibody against its cognate tag was determined as described in Fig. 1 (mean ± SEM; *n* = 3 to 5 independent experiments). (A) In paraformaldehyde‐fixed cells, three antibodies generated weak specific signals (AI177, AF371, and AI179). (B) In methanol‐fixed cells, the specific signals generated by different antibodies were highly similar to those obtained in paraformaldehyde‐fixed cells except for anti‐myc antibodies, which generated much weaker specific signals.

The normalized signal for each antibody/tag was determined in at least three independent experiments, using a high concentration of anti‐tag antibodies (5 μg·mL^−1^). An irrelevant antibody (AN703) was used to determine nonspecific binding of antibodies to cells. In paraformaldehyde‐fixed cells, all antibodies generated a signal at least twice higher than the background signal (Fig. 2A), indicating that they specifically recognize their cognate tag. Three antibodies (anti‐DYKDDDDK AI177, anti‐6xHis AF371, and anti‐myc AI179) generated low signals (< 25), while all others generated high specific signals (> 50; Fig. 2A).

When cells were fixed with methanol, the same overall hierarchy was seen, with two exceptions: in these conditions, both anti‐myc antibodies generated a much lower specific signal than in paraformaldehyde‐fixed cells (Fig. 2B); conversely, the signal generated by the anti‐SPOT antibody was increased. This result presumably reflects the fact that the conformations of the myc and SPOT epitopes slightly differ following paraformaldehyde and methanol fixation, a difference that alters their recognition by the antibodies tested in this study.

### Effect of antibody dilution on signal intensity

**Fig. 3:**
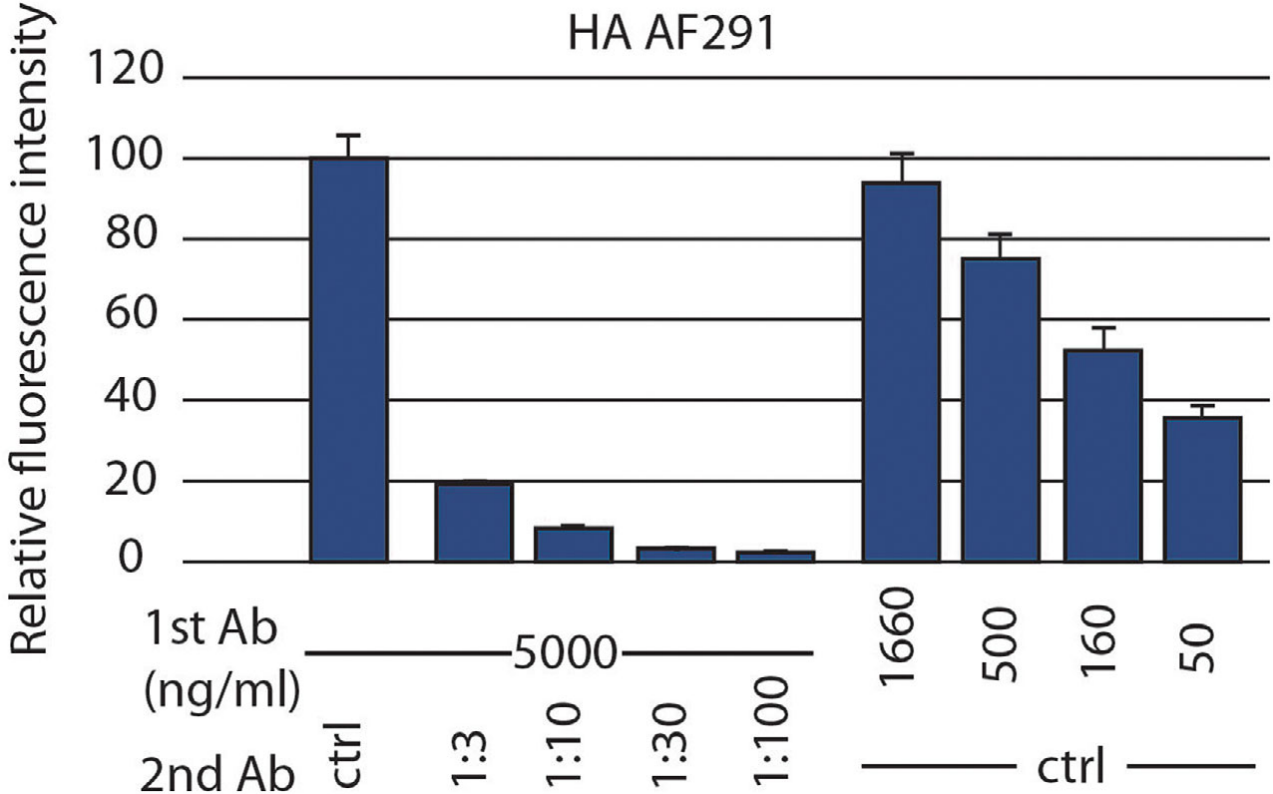
Amount of secondary but not primary antibody is limiting in our experimental setup. To assess whether primary or secondary antibodies were present in limiting amounts in our immunofluorescence procedure, we tested four dilutions of the primary (AF291) or of the secondary antibodies in parallel. The experiment was carried out as described in the legend to Fig. 2, with paraformaldehyde‐fixed cells (mean ± SEM; *n* = 50 cells). The signal decreased strongly when the concentration of the secondary antibody was diminished, but more gradually when the concentration of the primary anti‐tag antibody was decreased. This indicates that an excess of antibody is present when primary antibodies are used at a concentration of 5 μg·mL^−1^.

Several anti‐tag antibodies analyzed in this study generated comparable (high) specific signals, suggesting that, in the conditions used, either the primary (anti‐tag) or the secondary (fluorescent anti‐mouse Ig) antibodies may be present in limiting amounts. To investigate this possibility, we performed immunofluorescence in parallel in paraformaldehyde‐fixed cells with several dilutions of the anti‐HA AF291 antibody and with several dilutions of the secondary antibody. Our results clearly indicate that in our experimental setup the amount of secondary antibody was limiting: A dilution of the secondary antibody caused an approximately proportional decrease in the observed signal (Fig. 3). On the contrary, when the concentration of the primary antibody was reduced, this resulted in a relatively minor loss of signal: a 1:100 dilution of the primary anti‐HA antibody caused only a 60% decrease of the specific signal (Fig. 3). This result suggests that in this assay the anti‐HA antibody is used at a concentration much higher than necessary to obtain a high specific signal.

### A refined hierarchy of anti‐tag antibodies

**Fig. 4:**
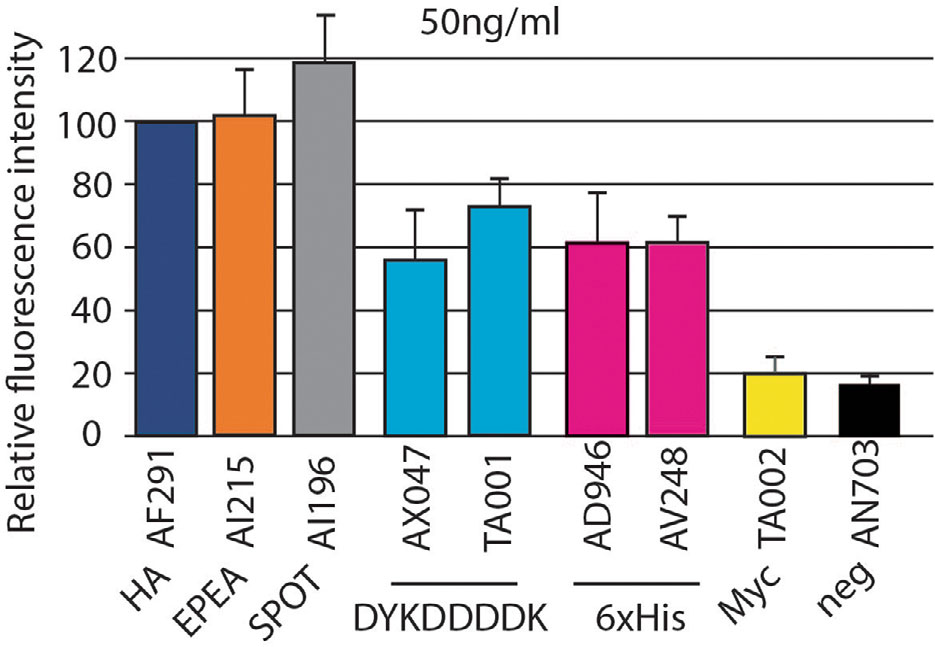
Detection of epitope tags in stringent conditions. In order to test antibodies in more stringent conditions, the efficiency with which each antibody bound its cognate tag in paraformaldehyde‐fixed cells was tested at low antibody concentration (50 ng·mL^−1^). In these conditions, all tested antibodies still exhibited specific binding, except the TA002 anti‐myc antibody (mean ± SEM; *n* = 3 independent experiments).

Although they generate similar signals at high concentrations, different anti‐tag antibodies may actually bind their cognate tag with different efficiencies. In order to test anti‐tag antibodies in more stringent conditions, we repeated the experiments described in Fig. 2 in paraformaldehyde‐fixed cells, using 100‐fold diluted solutions of anti‐tag antibodies (50 ng·mL^−1^; Fig. 4). Only antibodies generating a high signal at high concentrations were tested in these experiments. Some antibodies generated an intense signal even when diluted (AF291, AI215, and AI196), others (TA001, AX047, AD946, and AV248) were slightly less efficient. In these conditions, TA002 did not generate a specific signal.

## Discussion

In this study, we conducted a side‐by‐side quantitative comparison of the recognition of several epitope tags by their corresponding antibodies in immunofluorescence experiments. Three groups of epitopes/antibodies emerge: ‘good’, ‘fair’, and ‘mediocre’. While all antibodies generate a specific signal against their cognate tag, some ‘mediocre’ antibodies (e.g., AF371) generate a relatively low signal even when used at a high concentration. While ‘good’ antibodies (e.g., AF291) also generate specific signals when used at low concentrations, ‘fair’ antibodies (e.g., TA002) do not. Our results allow a quantitative comparison between different antibodies recognizing the same tag. For example, of the three antibodies tested, AI177 bound inefficiently the DYDDDDK epitope. This result is consistent with a previous report study where AI177 failed to generate a specific signal against a DYDDDDK‐tagged protein [11]. On the contrary, AX047 and TA001 recognize efficiently the DYDDDDK epitope. Overall, when using HA, EPEA, SPOT, DYKDDDDK, and 6xHis epitopes, our study suggests that optimal results will be obtained using antibodies AF291, AI215, AI196, TA001, and AV248, respectively. In our experimental conditions, all five antibodies generated similar signals, suggesting that none of them was clearly preferable to the others. In limiting conditions (50 ng·mL^−1^), antibodies against DYKDDDDK (TA001) and 6xHis (AV248) epitopes generated slightly less signal than antibodies against HA, EPEA, and SPOT. It remains to be seen whether these relatively small differences are significant or rather reflect more trivial experimental variability. It is sometimes believed that rationally designed epitopes (like DYKDDDDK) should be preferred to natural epitopes (like HA), or that recently developed antibodies (like AI215 or AI196) generate more or more specific signals than previously selected antibodies (like AF291). Our results do not support these notions. For practical purposes, it is worth noting that, among these antibodies, only AF291, TA001, and AV248 are devoid of intellectual property, allowing them to be produced and used with no restrictions. For researchers initiating a new project, the use of the HA or DYKDDDDK tags appears as a good choice.

We did not identify a ‘good’ antibody against the myc epitope. Unless a better antibody can be identified, it may be best to avoid using this epitope for immunofluorescence detection. If this is not possible, the TA002 is the best option tested in this study, but it should rather be used at a high concentration. In addition, our results indicate that methanol fixation may strongly reduce the specific signal of TA002.

Although our study provides a useful quantitative side‐by‐side comparison, its limitations should be kept in mind. First, our results are only valid in a precise experimental setting: immunofluorescence detection of proteins in paraformaldehyde‐ or methanol‐fixed cells. An antibody generating a low or null signal in a specific experimental setting (e.g., immunofluorescence) may prove valuable in another setting (e.g., immunocytochemistry and western blot). Second, we only analyzed a few antibodies recognizing a few epitope tags. A more extensive comparison may bring to light better antibodies. Third, the format of the antibodies (single‐chain minibodies, complete two‐chain antibody…), as well as the method used to produce them (mammalian cells and bacteria) may influence their binding properties. We only tested the minibody format in this study, where the antigen‐binding domain is fused to the Fc domain of choice. Fourth, we have chosen a configuration where the epitope tags are placed at the C terminus of the IL2Ra protein, in a position where they cannot be masked by the folding of the protein. It remains to be seen how efficiently each antibody recognizes its cognate tag when it is inserted at different positions in the protein of interest. With these limitations in mind, to our knowledge our study provides the first side‐by‐side quantitative comparison of different epitope tags/antibodies and can be used by researchers as a guide to choose optimal tags and antibodies.

## Methods

### Cell culture and reagents

Human embryonic kidney 293T cells were grown at 37 °C and 5% CO_2_ in Dulbecco's modified Eagle's medium (Gibco, Billings, MT, USA) containing 10% fetal bovine serum (Gibco) and 100 μg·mL^−1^ of penicillin–streptomycin (Sigma).

To express chimeric proteins, we used a previously described pCDM8‐based vector containing the coding sequence of the extracellular domain of the α chain of the interleukin‐2 receptor (human IL2Ra; amino acids 22 to 240, Uniprot P01589) and the sequence coding for the indicated epitope at the C‐terminal end of the cytosolic domain (Table 1).

All recombinant antibodies used in this study are listed in Table 1. AI215 (anti‐EPEA), AI196 (anti‐SPOT), AI179 (anti‐Myc), AF291 (anti‐HA), AF371, AD946, AV246 (anti‐6xHis), AI177, AX047 (anti‐Flag), AJ519 (anti‐TAC), and AN703 (negative control) were described previously (Table 1). TA001 and TA002 antibodies were generated in the frame of this study: TA001 was obtained by grafting the CDR regions of AW448 on a DP47/DPK22 frame [19] and the TA002 antibody by grafting the CDR regions of AF166 in a V_H_AF291‐V_L_AI830 frame. All antibodies are designated in this study with the unique identifiers provided by the ABCD database (https://web.expasy.org/abcd/) and were produced by the Geneva Antibody Facility as minibodies with the single‐chain variable fragment (scFv) or Variable Heavy chain of nanobodies (VHH) antigen‐binding portions fused to the indicated Fc region (https://www.unige.ch/medecine/antibodies/, https://abcd‐antibodies.com).

### Intracellular staining of tagged proteins

To express various tagged proteins, cells were transfected 2 days before the experiment using polyethylenimine as previously described [20].

Cells were then fixed for 30 min at room temperature in PBS containing 4% paraformaldehyde, then washed with PBS containing 40 mm NH_4_Cl, and permeabilized for 5 min in PBS containing 0.2% saponin. Alternatively, cells were fixed and permeabilized by an incubation for 2 min in methanol at −20 °C, followed by one wash in PBS. After blocking for 5 min with PBS containing 0.2% BSA (PBS‐BSA), fixed and permeabilized cells were then incubated for 30 min at room temperature in PBS‐BSA and the indicated primary antibodies at a concentration of 5000 (Figs 1 and 2) or 50 (Fig. 4) ng·mL^−1^. After three washes with PBS‐BSA, cells were further incubated for 30 min with fluorescent secondary antibodies: Alexa‐Fluor‐647‐coupled anti‐mouse‐immunoglobulin G (IgG) antibodies (Life Technologies; Carlsbad, CA, USA, A21235) and Alexa‐Fluor‐546‐coupled anti‐rabbit‐IgG antibodies (Life Technologies, A11030). Unless otherwise specified (Fig. 3), secondary antibodies were used at the concentration recommended by the manufacturer (1 : 400 dilution). Cells were then washed three times with PBS‐BSA, once with PBS and mounted in Möwiol. Pictures were taken using a Zeiss LSM700 confocal microscope, Oberkochen, Germany, with a 63× oil immersion objective.

### Signal quantification

The signals generated by each anti‐tag antibody and by the anti‐IL2Ra antibody were quantified with imagej software (http://rsb.info.nih.gov/ij/).

For each condition, three pictures were analyzed. In each picture, 20 regions of 30 × 30 pixels were selected and the mean gray value in the two fluorescence channels was measured.

The raw values obtained were plotted, and linear regression was used to calculate the efficiency with which the anti‐tag antibody recognized its epitope. For each antibody, the relative binding efficiency was compared with the value obtained for the AF291 antibody (set to 100), included in all experiments.

## Conflict of interest

PC and PH are cofounders and shareholders of ABCD Antibodies SA, a company dedicated to the production and distribution of research antibodies.

### Peer review

The peer review history for this article is available at https://www.webofscience.com/api/gateway/wos/peer‐review/10.1002/2211‐5463.13705.

## Author contributions

AM, WCL, and PH performed experiments. PC wrote the manuscript. All authors designed experiments, interpreted data, and read and corrected the manuscript.

## Data Availability

All processed data are available in the manuscript. All unprocessed experimental data will be made available online on a dedicated server of our University upon publication.
